# Identification of the minimal binding region of a *Plasmodium falciparum* IgM binding PfEMP1 domain

**DOI:** 10.1016/j.molbiopara.2015.06.001

**Published:** 2015-05

**Authors:** Jean-Philippe Semblat, Ashfaq Ghumra, Daniel M. Czajkowsky, Russell Wallis, Daniel A. Mitchell, Ahmed Raza, J.Alexandra Rowe

**Affiliations:** a Institute of Immunology and Infection Research, Centre for Immunity, Infection and Evolution, School of Biological Sciences, University of Edinburgh, Edinburgh EH9 3FL, United Kingdom; b Bio-ID Center, School of Biomedical Engineering, Shanghai Jiao Tong University, Shanghai, China; c Department of Infection, Immunity and Inflammation, University of Leicester, Leicester, United Kingdom; d Clinical Sciences Research Laboratories, Warwick Medical School, Coventry CV2 2DX, United Kingdom

**Keywords:** Rosetting, Cell Adhesion, Immunoglobulin M, Fc-receptor, Var gene, DBL domain

## Abstract

Binding of host immunoglobulin is a common immune evasion mechanism demonstrated by microbial pathogens. Previous work showed that the malaria parasite *Plasmodium falciparum* binds the Fc-region of human IgM molecules, resulting in a coating of IgM on the surface of infected erythrocytes. IgM binding is a property of *P. falciparum* strains showing virulence-related phenotypes such as erythrocyte rosetting. The parasite ligands for IgM binding are members of the diverse *P. falciparum* Erythrocyte Membrane Protein One (PfEMP1) family. However, little is known about the amino acid sequence requirements for IgM binding. Here we studied an IgM binding domain from a rosette-mediating PfEMP1 variant, DBL4ζ of TM284var1, and found that the minimal IgM binding region mapped to the central region of the DBL domain, comprising all of subdomain 2 and adjoining parts of subdomains 1 and 3. Site-directed mutagenesis of charged amino acids within subdomain 2, predicted by molecular modelling to form the IgM binding site, showed no marked effect on IgM binding properties. Overall, this study identifies the minimal IgM binding region of a PfEMP1 domain, and indicates that the existing homology model of PfEMP1-IgM interaction is incorrect. Further work is needed to identify the specific interaction site for IgM within the minimal binding region of PfEMP1.

## Highlights

• Many pathogens bind the Fc region of host immunoglobulin to evade immunity.
• We examined a *Plasmodium falciparum* IgM binding PfEMP1 domain TM284var1 DBL4ζ.
• We identified the minimal IgM binding region comprising subdomain 2 and flanking regions.
• Specific charged amino acids were mutated but did not markedly affect IgM binding.
• Existing models of PfEMP1-IgM interaction need to be revised.

## Graphical abstract

**Figure:**
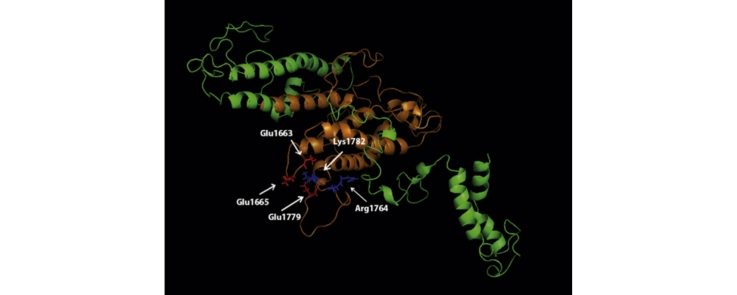

## 1 Introduction

Many pathogens have evolved to bind to a common site on the Fc portion of immunoglobulin, however, the consequences of such interactions are largely unexplored [1]. *Plasmodium falciparum*, the major cause of severe malaria, is an example of such a pathogen that has been shown to bind to the Fc region of human IgM [2]. Binding occurs during the asexual stage of the parasite life cycle on the surface of infected Red Blood Cells (iRBCs) to a parasite-derived ligand called *P. falciparum* Erythrocyte Membrane Protein 1 (PfEMP1) [2], which is a variant surface antigen encoded by the *var* gene family. Each parasite has approximately 60 *var* genes in its genome, with only one transcribed at a time per iRBC [3]. Switching of *var* gene transcription leads to a change in the PfEMP1 variant expressed on the surface of the iRBC and is responsible for antigenic variation of malaria parasites [3]. PfEMP1 molecules are made up of cysteine-rich adhesion domains called Duffy Binding Like (DBL) and Cysteine-rich Inter-Domain Regions (CIDR) that bind to a range of host receptors including CD36, Chondroitin Sulphate A (CSA), InterCellular Adhesion Molecule-1 and Endothelial Protein C Receptor [4]. The adhesion domains are further classified into subtypes, DBL (α, β, γ, δ, ϵ, ζ and *x*) and CIDR (α, β, γ, δ and PAM), based on sequence similarity [5,6]. Only a minority of PfEMP1 variants show IgM binding activity, but IgM binding is linked to several virulence-associated *P. falciparum* phenotypes such as rosetting with uninfected RBC in severe childhood malaria [7] and binding to CSA in placental malaria [8]. The molecular basis of IgM binding by PfEMP1 is not fully understood, but current data suggest that most IgM binding sites lie within specific DBLϵ and DBLζ domains [2,9–11].

**Fig. 1:**
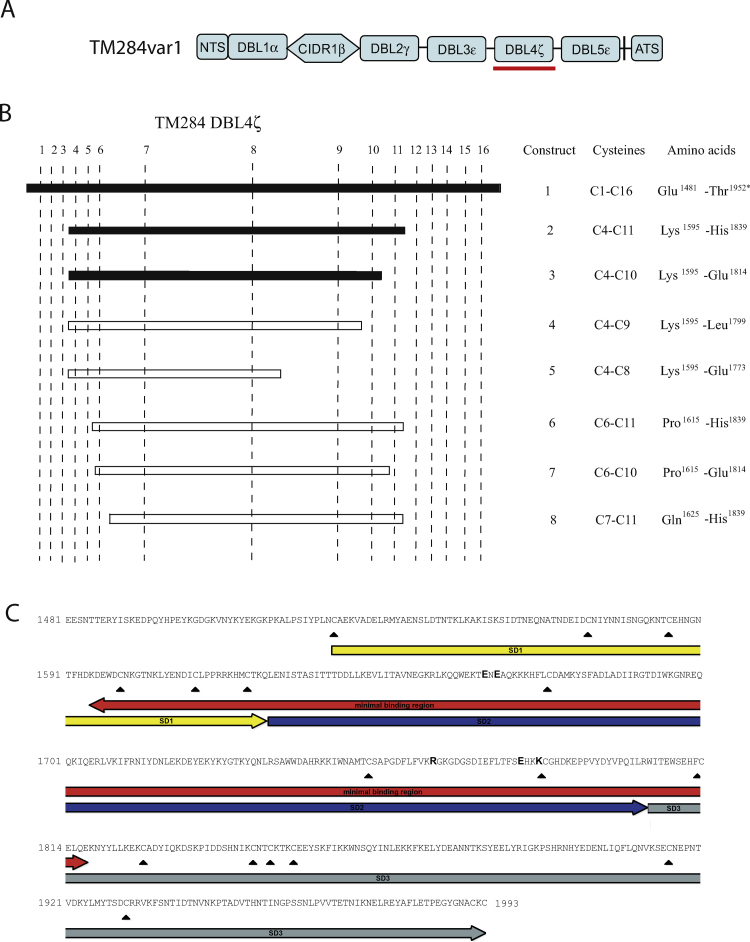
Identification of the minimal IgM binding region of the TM284var1 DBL4ζ domain. (A) Diagram showing the domain composition of the TM284var1 PfEMP1 variant, with the IgM binding DBL4ζ domain underlined in red. (B) Diagram showing the amino acid domain boundaries and IgM binding properties of each DBL4ζ deletion construct. The full length DBL4ζ domain (top bar) contains 16 cysteines as visualized by the dashed lines (construct used in previous work [2]). Seven deletion constructs were made spanning various regions of DBL4ζ. Proteins that bind human IgM are shown as black bars, and non-binding proteins are shown as white bars. (C) The minimal binding region of DBL4ζ (red). Subdomain (SD) 1 (yellow), 2 (blue) and 3 (grey) are shown, and cysteine residues are highlighted by arrowheads. The five charged residues within subdomain 2 predicted to be involved in IgM binding are shown in bold.

Previously we studied an IgM binding rosetting *P. falciparum* line TM284R+, which is a culture-adapted parasite derived from a Thai patient with cerebral malaria [12]. Rosetting is the binding of iRBC to two or more uninfected RBC, and is a PfEMP1-mediated parasite virulence phenotype that is implicated in severe malaria [13]. Many rosetting PfEMP1 variants bind IgM [14], and the IgM is thought to strengthen and stabilise the rosettes [12,15]. We identified the PfEMP1 variant expressed by IgM binding rosetting TM284R+ parasites as TM284var1, and showed that the IgM binding region is the fourth DBL domain from the N-terminus, DBL4ζ [2] (Fig. 1A). This domain was initially described as a DBLβ subtype, however, more recent analyses indicate that this domain is a DBLζ subtype [6]. Henceforth, we shall refer to this domain as TM284var1 DBL4ζ.

In our previous work, we localised the PfEMP1-IgM binding interaction site to the Cμ3-Cμ4 region of IgM Fc, and showed that the same site on IgM is used by multiple different *P. falciparum* genotypes [2,16]. Although, a common site on the host IgM molecule has been identified, the IgM binding site within a parasite DBL domain has not yet been investigated further. The aim of this study was to determine the minimal region within TM284var1 DBL4ζ required for IgM binding, and to use site-directed mutagenesis to investigate the role of specific amino acids within TM284var1 DBL4ζ identified as possible IgM-interaction sites from homology modelling.

## 2 Materials and methods

### 2.1 Deletion constructs and COS cell immunofluorescence assays

Deletion constructs based on TM284var1 (Genbank accession number JQ684046) DBL4ζ were amplified and cloned into the pRE4 vector as described previously [9,17]. The amino acid boundaries and primers used are shown in Table S1. Immunofluorescence assays (IFAs) were carried out as described previously [9]. Briefly, COS-7 cells were seeded in wells containing 12 mm coverslips and transfected with constructs using FuGene (Roche) according to the manufacturer’s protocol. IFAs were carried out forty-eight hours after transfection on cells that were washed with Phosphate Buffered Saline (PBS) and fixed for 10 min in PBS/2% formaldehyde. Cells were blocked for 1 h with PBS/5% Bovine Serum Albumin (BSA) and incubated with PBS containing 10 % pooled human serum as a source of IgM for 1 h. Cells were washed in PBS/0.1% BSA and incubated for 1 h with either a mouse anti-human IgM monoclonal antibody (mAb) (AbD Serotec MCA 1662) or mouse mAb DL6 (Santa Cruz Biotechnology sc-21719) diluted 1:1000 in PBS/0.1% BSA. DL6 detects the HSV-1 glycoprotein D tag at the C-terminal end of the cloned DBL domain [17]. Cells were washed in PBS/0.1% BSA and incubated with 1:6000 of Alexa Fluor 488-labelled goat anti-mouse IgG (Molecular Probes, A-11029) diluted in PBS/0.1% BSA for 45 min. Cells were washed for 10 min with PBS/0.1% BSA, mounted on a slide using Fluoromount-G (Southern Biotech) and viewed using a Leica DM LB2 fluorescence microscope. The transfection efficiency and/or IgM binding was assessed by counting the percentage of COS-7 cells showing positive surface fluorescence with DL6 or anti-IgM mAb in 10 fields with a 40*x* objective. The total number of COS-7 cells per field was counted using the auto-fluorescence of the cell nuclei to identify individual cells. The precise number of cells counted differed in experiments with varying cell confluence (80–100% confluent), but at least 500 COS-7 cells per slide were counted in all cases. Positive cells were defined as those showing fluorescence over the whole COS-7 cell surface as indicated in Fig. S1 and in our previously published work [2,9].

### 2.2 Molecular modelling

The homology model of TM284var1 DBL4ζ was constructed with the automated homology modelling tools in DeepView v.3.7 [18] using the structure of 3D7var2CSA DBL6ϵ (PDB accession code 2WAU) as the template, as described previously [16].

### 2.3 Expression and purification of mutant DBL4ζ protein

Site-directed mutagenesis of TM284var1 DBL4ζ was carried out by two-step PCRs using mutagenic primers and *Pfx* Platinum polymerase according to the manufacturer's protocol (Invitrogen). The primers used for the E1663R mutant were 5′-caatgggagaaaacacgaaatgaagcacaaaaa-3′ and 5′-tttttgtgcttcatttcgtgttttctcccattg-3′. The primers used for R1764E mutant were 5′-ttcctttttgtaaaagaaggaaaaggagatgga-3′ and 5′-tccatctccttttccttcttttacaaaaaggaa-3′. The first PCR amplified two fragments from the wild type construct that was used as a template. The two fragments contained overlapping complimentary ends and included the mutation that would result in an amino acid substitution. The second PCR used primers specific for the outer borders (used initially to PCR wild type DBL4ζ of TM284var1) to amplify the overlapping fragments made from the first PCR. These primers were 5′-aaggatccaactgtgctgaaaaggttgct-3′ (normal forward) and 5′- aagctagcttacttacatttacaagcattacc-3′ (normal reverse). The resulting PCR product was cloned into the pET15b-modified vector [19]. The E1663R, R1764E double amino acid mutant was generated using the E1663R construct as a template and PCR was carried out with primers shown above for R1764E. The resulting PCR product was used as a template for a second PCR with the normal forward and normal reverse primers and subsequently cloned into the pET15b-modified vector. The construct containing four mutations was generated in a similar manner as described above. Firstly, the R1764E construct was used as a template to introduce the E1665R and E1665R mutations by PCR using the 5′-caatgggagaaaacacgaaatcgagcacaaaaa-3′ and 5′-tttttgtgctcgatttcgtgttttctcccattg-3′ primers. The resulting PCR product containing E1663R, E1665R and R1764E mutations was used as a template to introduce the fourth E1779K mutation by PCR using the 5′-tttttaactttttcaaaacataaaaaatgtgga-3′ and 5′-tccacattttttatgttttgaaaaagttaaaaa-3′ primers and subsequently cloned into the pET15b-modified vector. DNA sequencing confirmed the presence of mutations in the resulting constructs and protein expression was performed as described earlier [20]. Briefly, *Escherichia coli* Origami B cells (Novagen) were transformed and grown to O.D. of 1.2 at 600 nm. Bacterial cultures were induced with a final concentration of 1 mM IPTG and grown overnight at 25 °C in an orbital shaker. Bacterial pellets were harvested, sonicated and protein was purified from soluble lysate using Ni-NTA metal affinity chromatography. Fractions containing protein were combined, concentrated using Amicon Ultra centrifugal filters (Millipore) and further purified by size exclusion chromatography on a Superdex 200 (16/60) column (GE Healthcare). Fractions were collected and the presence of protein at the expected molecular weight of approximately 58kDa was assessed by SDS-PAGE (see below). Fractions were concentrated and stored at −80 °C prior to use in circular dichroism (CD) or Surface Plasmon Resonance (SPR).

### 2.4 Characterisation of DBL4ζ mutants by SDS-PAGE and Western blot

The purity of the eluted protein was assessed by SDS-PAGE. Five μg of purified protein was prepared in loading buffer under non-reducing and reducing (5% β-mercaptoethanol) conditions and heated to 80 °C for 10 min. Five μl of broad range pre-stained marker (NEB) was run alongside the recombinant DBL4ζ proteins. Electrophoresis was carried out on 4–12% bis-Tris polyacrylamide gradient gels with MES SDS running buffer, and the gels were stained with Simply Blue SafeStain using the manufacturer’s protocols (Invitrogen). For the western blot, 5 μg wild type and mutant DBL domains were run on a 4–12% bis-Tris polyacrylamide gradient gel with MOPS SDS running buffer (Life Technologies). Replicate gels were either stained using Simply Blue SafeStain (Invitrogen) or transferred onto a PVDF membrane using the iBlot gel transfer device (Life Technologies). The membrane was probed with a Penta His HRP-conjugated antibody (1/2000; Qiagen), and developed using Super Signal West Pico (Thermo Scientific).

### 2.5 Characterisation of DBL4ζ mutants by circular dichroism (CD)

DBL4ζ recombinant proteins were dialysed in 50 mM phosphate buffer (pH 7.2) overnight at 4 °C and concentration adjusted to 0.1 mg/ml. CD spectra were recorded with 300 μl of DBL4ζ proteins using a Chiracan-plus spectrometer (Applied Photophysics) at 25 °C. A cell of 0.05 cm path length was used and measurements were recorded at 1 nm intervals between 190 and 260 nm at 1s averaging time for each point. Ten consecutive measurements were averaged and corrected against buffer alone. Results were normalized to mean molar differential coefficient per amino acid residue (http://dichroweb.cryst.bbk.ac.uk/html/userguide.shtml).

### 2.6 Surface Plasmon Resonance (SPR)

IgM (Calbiochem 401108) (25 μg/ml) was immobilized onto the surface of a GLC sensor chip (BioRad; ∼10,000 response units) at pH 5, using the manufacturer’s amine-coupling kit and protocol. The binding of DBL4ζ wild type and mutants to human IgM was measured using a ProteOn XP36 biosensor instrument (BioRad). Recombinant proteins in 10 mM Tris pH 7.4, containing 140 mM NaCl, 2 mM CaCl_2_ and 0.005% Tween-20 at 25 °C were tested over a range of concentrations (600, 300, 150, 75, 37.5 and 18.75 nM) at a flow rate of 25 μl/min. After each run the chip was regenerated with 10 mM Glycine-HCl (pH 2.5). The responses of specific binding to IgM-coated channels were calculated by subtracting the response obtained from binding to an uncoated lane monitored simultaneously. The sensograms were fitted using a 1:1 Langmuir kinetic model and the ProteOn Manager software was used to derive the values for *k*_a_, *k*_d_ and *K*_D_.

## 3 Results

### 3.1 Identification of the minimal IgM binding region of DBL4ζ (TM284var1)

We previously showed that recombinant TM284var1 DBL4ζ (amino acids Glu1481-Thr1952) containing 16 cysteine residues (construct 1) binds to human IgM [2]. We made deletion constructs based on this domain, which we expressed on the surface of COS-7 cells, to identify the minimal region that could bind IgM. Construct 2, containing 8 cysteine residues (C4-C11), and construct 3 containing 7 cysteine residues (C4-C10) bound IgM similarly to construct 1 (Fig. 1B and Table 1). Deleting cysteine 10 resulted in a loss of IgM binding (construct 4, C4–C9). The smaller constructs 5–8 also failed to bind IgM. Therefore, the minimal IgM binding region is C4–C10 (construct 3, Lys1595 to Glu1814).

**Fig. 2:**
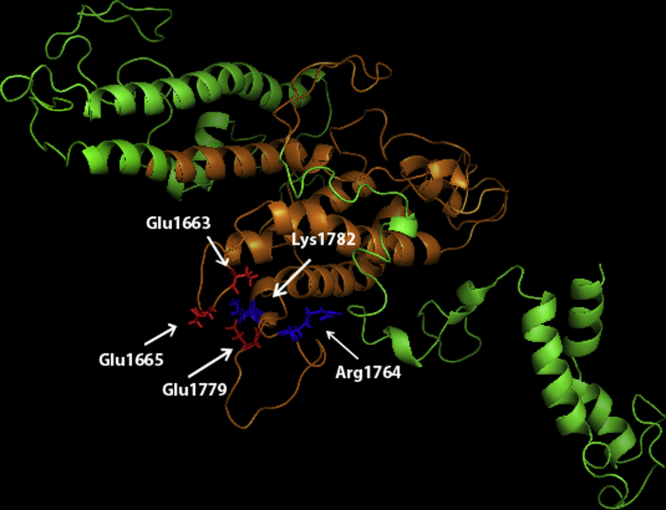
Homology model of TM284var1 DBL4ζ. The TM284var1 sequence starting from Glu1481 to Thr1952 was used to generate a model of DBL4ζ based on the structure of DBL6ϵ of 3D7var2CSA as described previously [16]. Amino acids 1595–1814, the minimal IgM binding region, are shown in orange. Negatively charged amino acids postulated to bind IgM are shown in red (Glu1663, Glu1665 and Glu1779, and positively charged residues in blue (Arg1764 and Lys1782).

DBL domains are composed of a core scaffold of alpha-helical bundles stabilised by disulphide bonds, and consist of three subdomains [21]. The minimal IgM binding region comprises all of subdomain 2 of TM284var1 DBLζ, with flanking parts of subdomain 1 and 3 (Fig. 1C). The minimal binding region is shown in orange in a homology model of the TM284var1 DBLζ domain (Fig. 2).

### 3.2 Production of TM284var1 DBL4ζ mutants to investigate the IgM binding site

We previously used homology modelling of the TM284var1 DBL4ζ domain [16] and human IgM [22] to construct a model of the DBL4ζ-IgM complex. The DBL-IgM docking model was generated under constraints that took into account the narrowness of the IgM Cμ3-Cμ4 interdomain region and the position and limited solvent accessibility of the DBL domain in the context of the entire PfEMP1 molecule [16]. In this model, the predicted interaction site included five charged amino acid residues in subdomain 2 of DBL4ζ (Fig. 2) that were immediately adjacent to oppositely charged residues in IgM, suggesting that these residues might be important for the interaction. The deletion mutant experiments in Fig. 1 confirmed that subdomain 2 is essential for IgM binding, consistent with a role for the five charged residues.

**Fig. 3:**
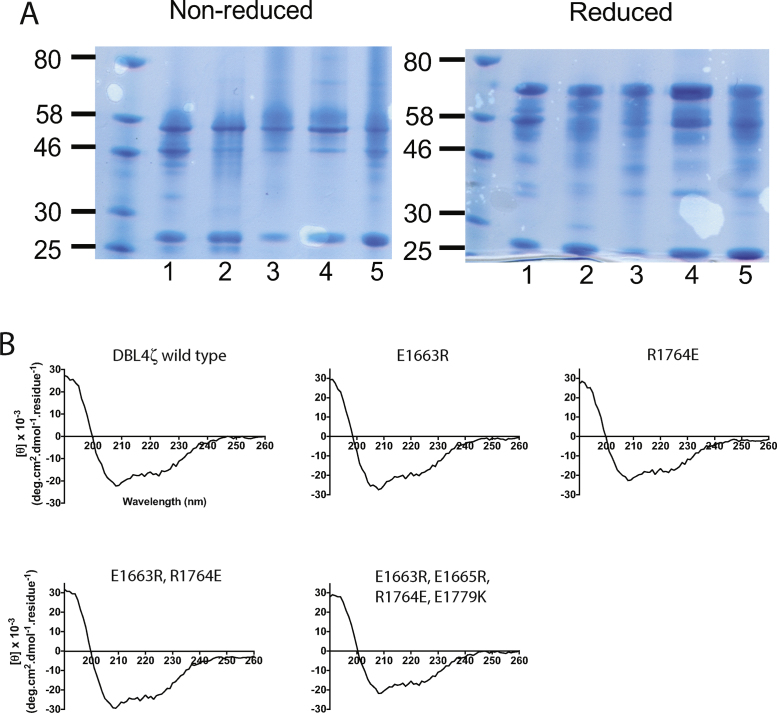
Characterization of recombinant DBL4ζ mutant proteins. (A) SDS-PAGE of purified wild type DBL4ζ and mutant proteins on 4–12% bis-Tris polyacrylamide gels. Five micrograms of proteins were used per lane and the broad range pre-stained protein marker (NEB) was used as a reference. Predominant bands at approximately 56 kDa were observed under non-reducing conditions, which shifted upon reduction, which is typical of DBL domains and characteristic of folded proteins with disulphide bonds. Lanes were as follows: (1) Wild type DBL4ζ, (2) E1663R, (3) R1764E, (4) E1663R, R1764E and (5) E1663R, E1665R, R1784E, E1779K. (B) CD spectra of purified recombinant wild type and mutant DBL4ζ proteins. Minima near 208 and 222 nm and maximum near 190 nm indicate the presence of significant α-helical content.

Therefore, to test the role in IgM binding of the charged residues predicted by the model, we expressed mutant recombinant proteins in *E. coli* and tested the ability of each mutant to bind human IgM. In each mutant the key residue(s) were mutated to residues showing the opposite charge. The proteins tested were single amino acid mutants E1663R and R1764E, double mutant E1663R, R1764E and quadruple mutant E1663R, E1665R, R1764E, E1779K. All proteins were expressed as soluble his-tagged proteins in *E. coli* and were purified using nickel affinity chromatography followed by size exclusion chromatography. The proteins were assessed by SDS-PAGE and in each case showed a major band about 56 kDa under non-reducing conditions, with a shift upon reduction consistent with the presence of disulfide bonds in the recombinant proteins (Fig. 3A). All proteins showed complex size exclusion chromatograms (an example is shown in Fig. S2) and contained some smaller fragments in addition to the major species (Fig. 3A), possibly due to proteolytic degradation [23]. Western blotting with an anti-his mAb showed that all the proteins of approx. 50 kDa and larger retained the N-terminal his-tag. However, the 27 and 23 kDa fragments were not recognised by the anti-his mAb, and therefore could be bacterial impurities or DBL fragments cleaved at the N-terminus (Fig. S3).

We assessed the quality of the expressed proteins by circular dichroism to test whether the recombinant DBL4ζ mutants and wild type are comparable and show the characteristic secondary structure seen with other DBL proteins. All four mutants showed a characteristic α-helical signature at 208 and 222 nm, similar to the wild type protein (Fig. 3B). Deconvolution of the CD spectra using CDNN software (Applied Photophysics) showed similar distribution of secondary structure components for each recombinant DBL4ζ protein (Table 2), suggesting that the mutants show similar overall DBL folds to the wild type. Furthermore, the CD spectra and secondary structure predictions are highly similar to those reported previously for other DBL recombinant proteins [24,25].

### 3.3 Binding of recombinant TM284var1DBL4ζ mutants to human IgM

**Fig. 4:**
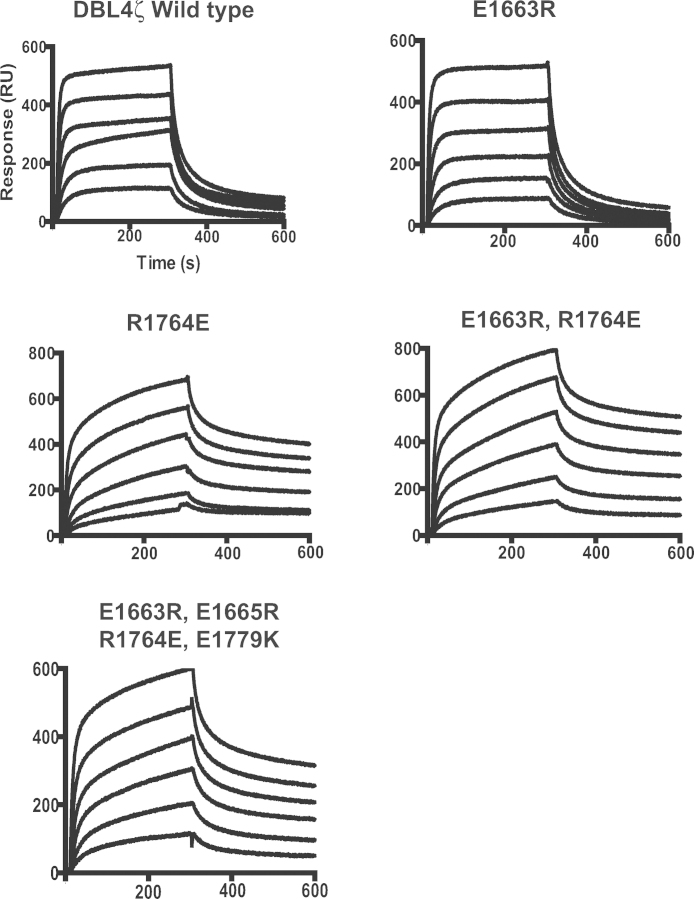
Binding of DBL4ζ mutants to human IgM by SPR. SPR sensograms showing wild type and mutant DBL4ζ recombinant proteins binding to human IgM. Proteins were diluted two-fold starting at 600 nM. All six dilutions were flowed at the rate of 25 μl/min (200 s contact time and 200 s dissociation) and regenerated with Glycine-HCl (pH 2.5). Values obtained using an uncoated lane were subtracted to give specific binding data. All of the recombinant proteins that contain the R1764E mutation did not fully dissociate, suggesting that some protein may be interacting non-specifically to the chip. Two independent experiments were carried out with similar results.

Surface Plasmon Resonance (SPR) was used to assess the binding of single (E1663R and R1764E), double (E1663R, R1764E) and quadruple (E1663R, E1665R, R1764E, E1779K) mutants to immobilized IgM. All mutants bound IgM similarly to the wild type (Fig. 4), with binding affinities (*K*_D_) in the nanomolar range for all proteins (Table 3). There were minor differences in *K*_D_ between some of the proteins, but further investigation with completely pure protein preparations would be required to determine the significance of these minor changes. All mutants containing R1764E showed biphasic association and dissociation curves (Fig. 4). Fitting the data to a model with two independent binding sites gave a *K*_D_ similar to the value obtained for the single-site model, together with a much tighter complex with no detectable dissociation over the time course of the experiment (koff < 10^−6^ s^−1^, the lower limit of the SPR machine). This suggests that a fraction of the R1764E protein preparation bound irreversibly to the chip, probably due to small amounts of aggregates. Critically, however, the rest of the protein bound with similar kinetics to the wild type protein. Overall, the SPR data show that the mutated residues do not play a major role in binding human IgM, although further work would be needed to exclude minor effects.

## 4 Discussion

In this study, we identified the minimal IgM binding region of the DBL4ζ domain of TM284var1, and tested the function of amino acid residues predicted from a PfEMP1-IgM homology model to be involved in binding IgM. We used deletion mutants to identify the minimal IgM binding region comprising all of subdomain 2 of DBL4ζ, along with adjoining regions of subdomains 1 and 3, containing seven cysteine residues in total. However, testing of charged amino acids within subdomain 2 that were predicted from homology modelling to form part of the interaction site with IgM, showed no large effect on IgM binding when the specific amino acids were mutated to ones bearing the opposite charge. Therefore the existing model is not supported by experimental data, and needs to be revised.

Recent structural studies of DBL-receptor complexes have shown that although the DBL core scaffold composed of alpha helices is very similar between different DBL structures, there is extensive variation in the surface exposed loops linking the helices [21,26–28]. Many of the known receptor binding sites map to such variable loops [21,26–28]. This diversity may limit the use of homology models to predict binding residues that are present in variable loops. X-ray crystallography provides the gold standard for identifying contact residues between receptors and ligands. However, this approach is currently problematic for the study of DBL-IgM interactions, due to the large size of IgM.

One of the limitations of the current study was the difficulty in preparing completely pure recombinant protein. Even after size exclusion chromatography, multiple protein species were seen on SDS-PAGE. Despite this, the DBL4ζ proteins showed the characteristic secondary structure of DBL domains by CD, and all proteins bound human IgM at nanomolar concentrations by SPR. Western blotting indicated that the >50 kDa proteins were his-tagged, likely representing the full-length protein and fragments with limited proteolytic degradation at the C-terminus. The experiments shown in Fig. 1 indicate that up to 138 amino acids (17 kDa) can be removed from the C-terminus of the construct without loss of IgM binding, therefore it is likely that all of the DBL4ζ fragments in the 50–65 kDa range contributed to the IgM binding interactions determined by SPR. Smaller fragments at ≈25 kDa protein were also present, which were not his-tagged, and could either represent bacterial impurities or DBL4ζ fragments that were degraded at the N-terminus. In either case, it is unlikely that these small fragments contributed to the IgM binding measure by SPR. This could lead to a slight underestimation of the binding affinity measured here, because the true concentration of the DBL4ζ protein in the assay is less than the apparent concentration used to calculate the *K*_D_ values. Despite the uncertainty introduced by the lack of completely pure protein, it is clear from the SPR data that all the mutant proteins retained the ability to bind human IgM at similar concentrations as the wild type. Repeated experiments with more highly purified protein preparations would be needed to determine whether there are any minor differences in binding affinity between the mutants and the wild type.

Recent work has provided some insights into the function of PfEMP1-IgM interactions. The binding of IgM to the VAR2CSA PfEMP1 variant, that plays a key role in sequestration of iRBC in the placenta in pregnancy malaria, has been shown to be an immune evasion mechanism that masks the iRBC from being targeted by parasite-specific IgG antibodies [29]. Other studies have shown that IgM binding enhances cell–cell adhesion in the context of rosette formation [10,15]. Whether IgM binding by *P. falciparum* proteins also results in effects on host Fcμ receptors or B cell receptors remains unknown [16]. Further work is needed to gain an accurate understanding of the molecular basis of DBL-IgM binding interactions and their influence on *Plasmodium* host-parasite interactions and severe malaria.

## Figures and Tables

**Table 1:** Summary of transfection efficiency and IgM binding of TM284var1 DBL4ζ deletion constructs expressed in COS-7 cells.

Construct	Cysteines	Transfection efficiency^a^ (%)	IgM binding^b^ (%)
1^c^ E1481-T1952	C1–C16	10–15	8–12
2 K1595-H1839	C4–C11	15–20	10–15
3 K1595-E1814	C4–C10	25–30	10–15
4 K1595-L1799	C4–C9	20	0
5 K1595-E1773	C4–C8	15–20	0^d^
6 P1615-H1839	C6–C11	10–15	0^d^
7 P1615-E1814	C6–C10	10–15	0
8 Q1625-H1839	C7–C11	15–20	0

a Transfection efficiency was determined by counting the percentage of COS-7 cells showing surface fluorescence by IFA with mAb DL6.

b IgM binding was determined by counting the percentage of COS-7 cells showing surface fluorescence by IFA using a mouse anti-human IgM mAb. Data from at least 2 experiments for each construct is shown.

c Construct used in previous study [2].

d Some faint positive cells (1–2%) were seen with these constructs in some but not all experiments (*n *= at least 3).

**Table 2:** Secondary structure elements in wild type and mutant proteins predicted from circular dichroism.

Protein	α-Helices (%)	α-Parallel β-sheets (%)	Parallel β-sheets (%)	β-Turns (%)	Random-coils (%)	Total secondary elements (%)
DBL4ζ wild type	52	3.0	4.5	14.8	15.3	89.6
E1663R	58.2	2.6	3.4	14.4	9.8	88.4
R1764E	54.3	2.4	4.3	14.3	14.5	89.9
E1663R, R1764E	68.9	0.8	2.7	12.3	9.3	93.1
E1663R, E1665R, R1764E, E1779K	59.3	2.2	4.6	14.2	16.1	91.0

**Table 3:** Association rate (*k*_a_), dissociation rate (*k*_d_) and binding affinity (*K*_D_) for DBL4ζ recombinant proteins.

Recombinant protein	*k*_a_ (M^−1^ s^−1^)	*k*_d_ (s^−1^)	*K*_D_ (nM)
Wild type	1.79 × 10^5^	1.18 × 10^−2^	66.2
E1663R	1.29 × 10^5^	1.48 × 10^−2^	115.0
R1764E	4.51 × 10^4^	1.89 × 10^−3^	41.6
E1663R, R1764E	4.94 × 10^4^	1.59 × 10^−3^	33.2
E1663R, E1665R, R1764E, E1779K	6.16 × 10^4^	2.54 × 10^−3^	41.2
