# Supplementary material for: Identification of the minimal binding region of a Plasmodium falciparum IgM binding PfEMP1 domain
Source: Mol Biochem Parasitol. 2015 May;201(1):76–82. doi: 10.1016/j.molbiopara.2015.06.001 (PMC4539346; doi:10.1016/j.molbiopara.2015.06.001)
Supplement: Supplementary file 1 [file mmc1.pdf]

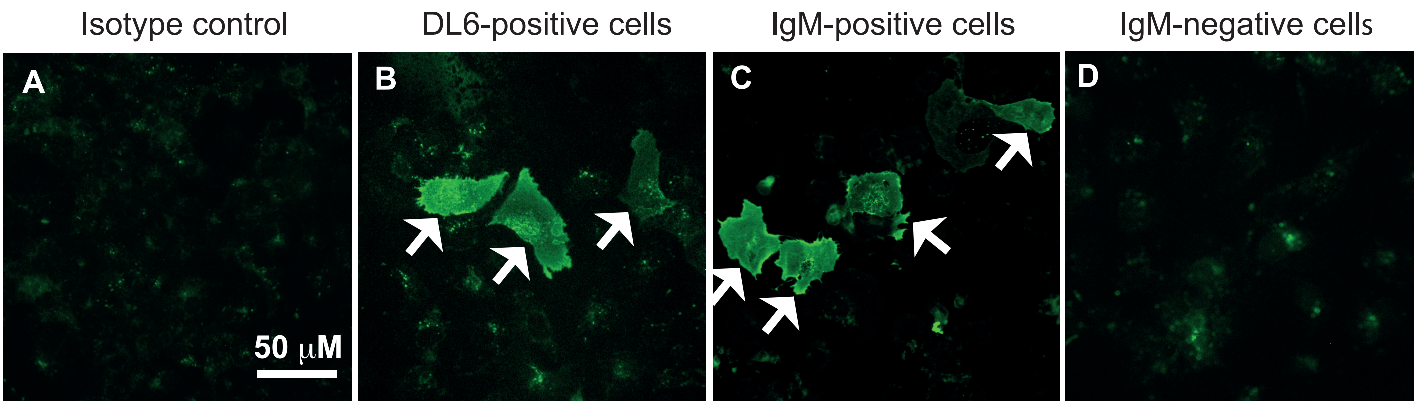

Figure S1. Representative images from COS-7 cell immunofluorescence assays. Transfected COS-7 cells were stained with mAb DL6 to detemine transfection efficiency or with anti-human IgM mAb to determine IgM-binding. An isotype control (A) shows no specific fluorescence over the COS-7 cells. DL6-positive (B) and IgM-positive (C) cells are detected by fluorescent staining over the entire surface of individual COS-7 cells (white arrows). Non-IgM-binding transfectants (D) show a similar appearance to the isotype control. All images taken at the same magnification and exposure settings.
