# Supplementary material for: Identification of the minimal binding region of a Plasmodium falciparum IgM binding PfEMP1 domain
Source: Mol Biochem Parasitol. 2015 May;201(1):76–82. doi: 10.1016/j.molbiopara.2015.06.001 (PMC4539346; doi:10.1016/j.molbiopara.2015.06.001)
Supplement: Supplementary file 2 [file mmc2.pdf]

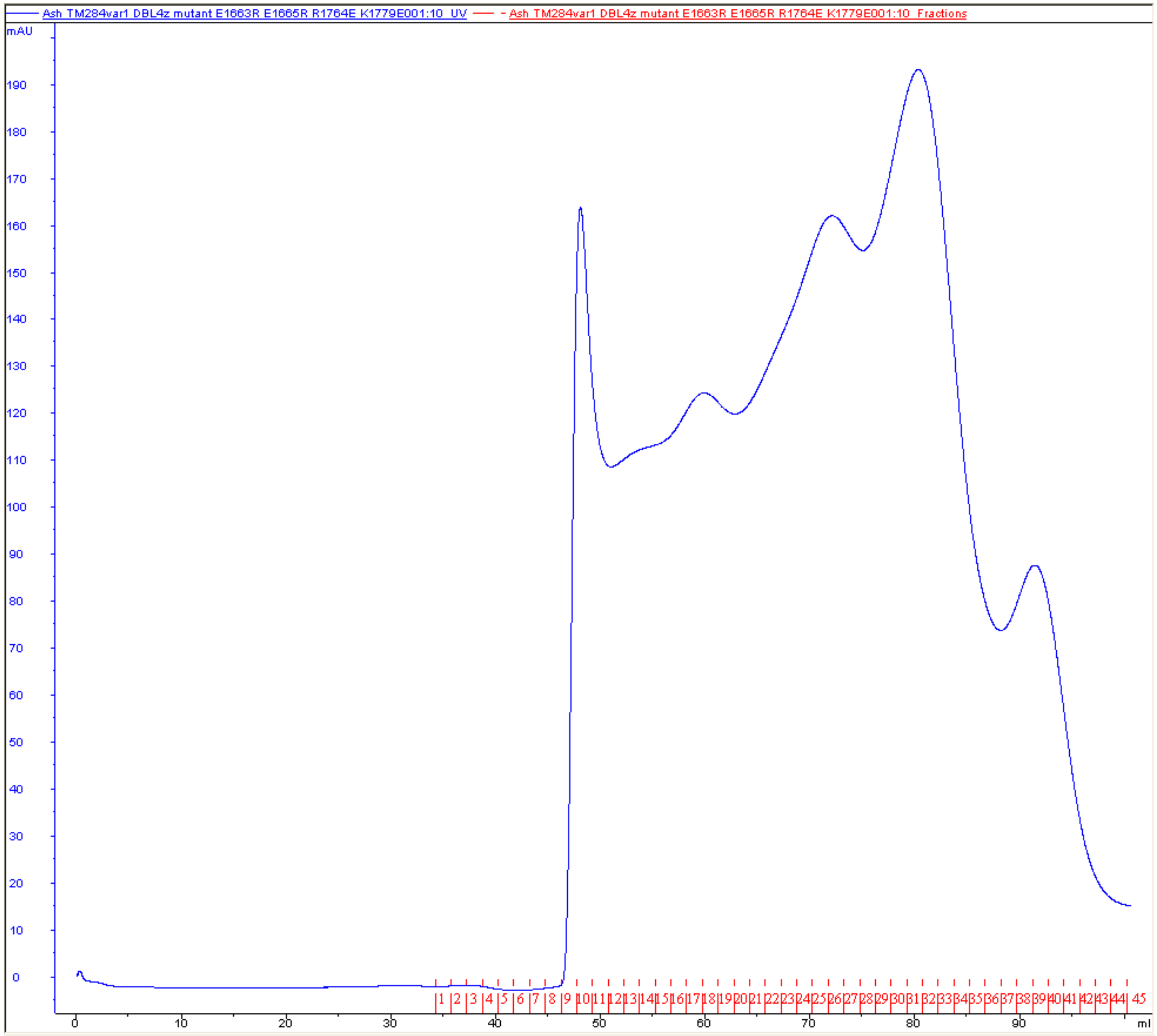

Figure S2. Size exclusion chromatogram for TM284var1 DBL4ζ mutant E1663R/E1665R/R1764E/K1779E. Fractions 28-32 were pooled and used for experiments. All fractions contained multiple bands by SDS-PAGE (eg. Figure 3A).
