# Supplementary material for: Identification of the minimal binding region of a Plasmodium falciparum IgM binding PfEMP1 domain
Source: Mol Biochem Parasitol. 2015 May;201(1):76–82. doi: 10.1016/j.molbiopara.2015.06.001 (PMC4539346; doi:10.1016/j.molbiopara.2015.06.001)
Supplement: Supplementary file 3 [file mmc3.pdf]

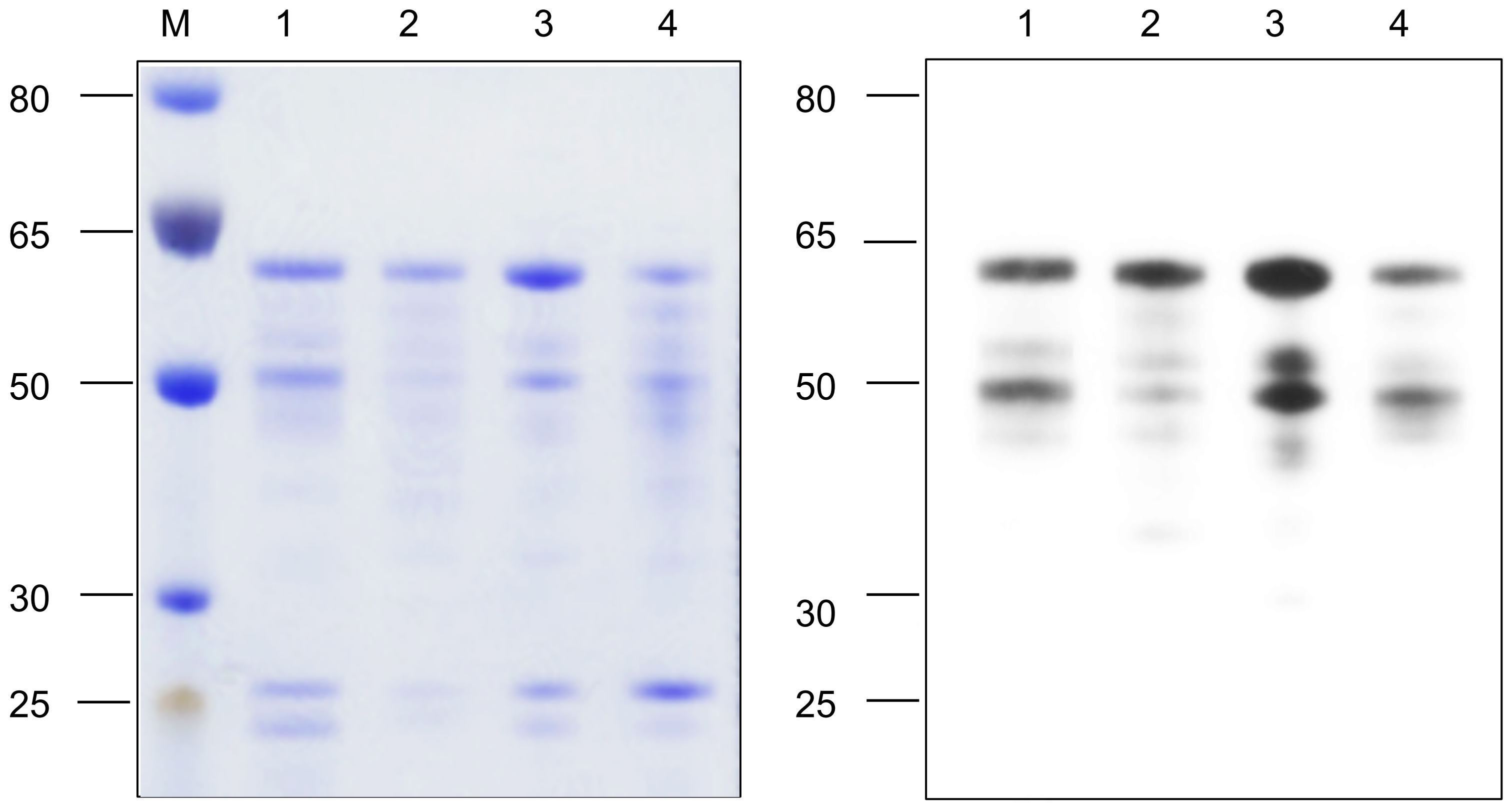

Figure S3. SDS-PAGE and Western-blot of TM284var1 DBL4ζ recombinant proteins. A) SDS PAGE and B) Western-blot using anti-His antibody. M: Page Ruler ™ Plus pre-stained protein ladder (Thermo Scientific); lane 1: DBL4ζ wild type; lane 2: DBL4ζ R1764E; lane 3: DBL4ζ E1663R/R1764E and lane 4: DBL4ζ E1663R, E1665R, R1764E, K1779E.
