# Supplementary material for: Identification of the minimal binding region of a Plasmodium falciparum IgM binding PfEMP1 domain
Source: Mol Biochem Parasitol. 2015 May;201(1):76–82. doi: 10.1016/j.molbiopara.2015.06.001 (PMC4539346; doi:10.1016/j.molbiopara.2015.06.001)
Supplement: Supplementary file 4 [file mmc4.docx]

**Supplementary Table**

**Table S1.** Oligonucleotide primers used to make TM284var1 DBL4ζ deletion constructs.

| **Construct** | **Amino acid boundaries** | **Primer sequence (5’ to 3’)** |
| --- | --- | --- |
| 1* | Glu1481-Thr1952 | TCTCGTCAGCTGGAAGAGTCAAATACTACG  ACGAGTGGGCCCAGTAACATCCGCAGTAGG |
| 2 | Lys1595-His1839 | TCTCGTCAGCTGAAGGATGAATGGGATTGTAAC  ACGAGTGGGCCCATGTGAATCATCAATAGG |
| 3 | Lys1595-Glu1814 | TCTCGTCAGCTGAAGGATGAATGGGATTGTAAC  ACGAGTGGGCCCTTCTTGCAATTCACA |
| 4 | Lys1595-Leu1799 | TCTCGTCAGCTGAAGGATGAATGGGATTGTAAC  ACGAGTGGGCCCAAGTATTTGAGGCACATAATC |
| 5 | Lys1595-Glu1773 | TCTCGTCAGCTGAAGGATGAATGGGATTGTAAC  ACGAGTGGGCCCTTCGATGTCACTTCCATCTCC |
| 6 | Pro1615-His1839 | TCTCGTCAGCTGCCTCCAAGAAGAAAACATATG  ACGAGTGGGCCCATGTGAATCATCAATAGG |
| 7 | Pro1615-Glu1814 | TCTCGTCAGCTGCCTCCAAGAAGAAAACATATG  ACGAGTGGGCCCTTCTTGCAATTCACA |
| 8 | Gln1625-His1839 | TCTCGTCAGCTGCAACTAGAAAATATCAGCACG  ACGAGTGGGCCCATGTGAATCATCAATAGG |

The restriction enzyme sites incorporated into the primers to facilitate cloning into pRE4 are underlined. *Construct used in previous study [2].
